# Decoding Salivary ncRNAomes as Novel Biomarkers for Oral Cancer Detection and Prognosis

**DOI:** 10.3390/ncrna11020028

**Published:** 2025-03-20

**Authors:** Subhadeep Das, Sampad Basak, Soumyadev Sarkar

**Affiliations:** 1 Department of Biochemistry, Purdue University, BCHM A343, 175 S. University Street, West Lafayette, IN 47907-2063, USA; 2 Purdue University Institute for Cancer Research, Purdue University, Hansen Life Sciences Research Building, Room 141, 201 S. University Street, West Lafayette, IN 47907-2064, USA; 3 Gujarat Biotechnology University, Gujarat International Finance Tec-City, Gandhinagar 382355, Gujarat, India; basaksampad0@gmail.com; 4 Center for Fundamental and Applied Microbiomics, Biodesign Institute, Arizona State University, Tempe, AZ 85281, USA

**Keywords:** oral cancer, non-coding RNA, biomarker, saliva

## Abstract

Oral cancer (OC) ranks among the most prevalent head and neck cancers, becoming the eleventh most common cancer worldwide with ~350,000 new cases and 177,000 fatalities annually. The rising trend in the occurrence of OC among young individuals and women who do not have tobacco habits is escalating rapidly. Surgical procedures, radiation therapy, and chemotherapy are among the most prevalent treatment options for oral cancer. To achieve better therapy and an early detection of the cancer, it is essential to understand the disease’s etiology at the molecular level. Saliva, the most prevalent body fluid obtained non-invasively, holds a collection of distinct non-coding RNA pools (ncRNAomes) that can be assessed as biomarkers for identifying oral cancer. Non-coding signatures, which are transcripts lacking a protein-coding function, have been identified as significant in the progression of various cancers, including oral cancer. This review aims to examine the role of various salivary ncRNAs (microRNA, circular RNA, and lncRNA) associated with disease progression and to explore their functions as potential biomarkers for early disease identification to ensure better survival outcomes for oral cancer patients.

## 1. Introduction

Oral squamous cell carcinoma (OSCC), commonly referred to as oral cancer (OC), is the most common form of head and neck cancer, originating in the tongue, lips, and floor of the mouth. It ranks among the top ten causes of cancer-related death rates globally [1,2,3]. In India, OSCC or OC are the most observed, accounting for almost one-third of the overall cases globally [3,4]. According to recently released statistics in GLOBOCAN, there were around 3,77,713 new cases of OC worldwide in 2020, while there were over 177,757 fatalities [1]. The prevalent risk factors for OSCC encompass betel quid chewing with areca nut, excessive tobacco use or smoking, alcohol intake, inadequate oral hygiene, and nutritional deficiencies. Additional factors include environmental effects, persistent viral infections such as human papillomavirus (HPV), and microbial infections [2,3,5]. OSCC originates from the mucosal epithelial cells found in the oral cavity [6,7]. The genetic features of OSCC exhibit complexity and significant heterogeneity [6,8]. Even with progress in understanding and scientific discoveries over the last two decades, the 5-year survival rate for oral cancer patients continues to fall short of 50% [3,9]. The disease’s aggressiveness and heterogeneity, coupled with late diagnosis, the absence of early detection markers, ineffective chemotherapeutic drugs, therapy resistance, and side effects, frequently hinder its management [2,10]. Despite some encouraging outcomes, EGFR-targeted treatment and PD-1/PD-L1 immune therapy, which have been approved by the US Food and Drug Administration (FDA), have had minimal success [2,11,12].

Recent advancements in high throughput genome sequencing have shown that over 98% of the human genome consists of non-coding transcripts that do not translate into any protein [2]. Long non-coding RNA (lncRNA) has garnered considerable interest among non-coding RNAs for its role as either a tumor suppressor or an oncogene. lncRNAs are considered biomarkers in oral pre-malignant lesions due to their over or under-expression and have been linked to tumor initiation, progression, and metastasis, along with drug resistance [3]. Similarly, circular RNA (circRNA) has been studied, potentially serving as a biomarker due to its ability to sponge miRNA thereby regulating gene expression. Various microRNAs (miRNAs) are examined for their multifaceted roles in the hallmarks of cancer and are considered potential biomarkers for cancer detection and prognosis [3]. RNAseq studies have identified over 200 ncRNAs (including miRNA, lncRNA, circRNA, snoRNA, and piRNA) linked to OC progression [2].

In OSCC, various investigators have observed abnormalities in different lncRNAs, including *MALAT1*, *HOTTAIR*, *HOTTIP*, and *TUG1* [6,13,14,15,16,17,18]. With its role as a tumor suppressor and tumor-promoting oncogenic driver, lncRNA might be the origin of the functional dysregulation linked to all pathophysiological abnormalities in OSCC [6]. *miRNA-145*, recognized as a significant tumor suppressor miRNA, is crucial in the regulation of apoptosis [19,20] and is often found to be downregulated in various cancers [20,21,22,23]. The downregulation of *miRNA-145* has been shown in an animal model of OSCC [24]. *miRNA-184* has the potential to target numerous genes and plays a role in inhibiting neuroblastoma cell survival by targeting AKT2, the serine/threonine kinase [25]. The Wong lab demonstrated that *miRNA-184* was crucial in the anti-apoptotic and proliferative events associated with OSCC [20,26].

Saliva, an isotonic fluid produced by the parotid, submandibular, and submaxillary salivary glands, bathes the oral cavity [27,28]. Saliva is a distinctive biological fluid characterized by a diverse range of proteins, polypeptides, nucleic acids, electrolytes, and hormones. The salivary glands produce a hypotonic exocrine secretion, exhibiting a pH range of 7.2 to 7.4 [29]. Saliva serves as the initial biological medium that interacts with external substances, protecting the mucosa of the upper digestive tract, particularly in the oral cavity and pharynx. Human saliva exhibits a total antioxidant capacity that surpasses that of blood plasma [30]. Saliva comprises polypeptides, immunoglobulin, and enzymes such as lactoferrin, lysozyme, and histamine. These polypeptides are essential in defense mechanisms against free radicals, preventing oral cancer [31,32]. Multiple agents induce carcinogenic effects by altering the biochemical composition of human saliva. Staterin is an acidic salivary protein that inhibits the accumulation of calcium phosphate in the salivary glands’ excretory ducts and modulates tooth enamel’s solubility [33,34,35]. The concentration of statherin in the saliva of patients with oral cancer is diminished, resulting in reduced function within the oral cavity [35,36].

Cystatins are proteins characterized by their chemical structure and function as inhibitors of the enzyme cysteine proteases. Cystatin SA-I, with a molecular weight of 14 kDa, has been identified in the saliva of patients diagnosed with oral squamous cell carcinoma. This protein exhibits greater levels in the saliva of patients prior to treatment than in the saliva of treated patients, suggesting its potential as a biomarker for oral squamous cell carcinoma [35,37]. Epidermal growth factor (EGF) is a protein crucial in regulating the balance of the oral mucosa and the mucosa of the upper gastrointestinal tract. It additionally promotes the healing of wounds in the oral environment.

The saliva of patients with oral cancer shows a reduced concentration of EGF, leading to a diminished potential for the renewal of the epithelium of the oral mucosa in these individuals [35,38,39]. Epithelial markers (CA125, CA19-9, tissue polypeptide antigen, carcinoembryonic antigen, CYFRA 21-1) are found in higher concentrations in the saliva of patients diagnosed with OSCC [35]. Matrix metalloproteinases (MMPs) are enzymes that play a role in developing oral cancer progression.

The uncontrolled activity of MMPs in tumor tissues primarily contributes to the degradation of proteins such as collagen, elastin, and fibronectin. The saliva of patients with OSCC has shown elevated MMP-2 and MMP-9 activity [35,40,41]. The composition and qualities of saliva change in response to the interactions between the environment, microbiome, and host response. Reports indicate that *P. gingivalis* infection significantly enhances the invasive potential of OSCC cells through the upregulation of IL-8 and MMPs [42]. *F. nucleatum* promotes the production of cytokines, including TNF, IL-6, 8, 10, and 12, as well as ROS and kinase, which enhance the progression of oral cancer [43,44]. Reactive oxygen species (ROS), including hydrogen peroxide and oxygen radicals, along with reactive nitrogen species (RNS) such as nitric oxides, reactive lipids, and metabolites like malondialdehyde and hydroxy-2-nonenal, together with MMPs produced by immune cells, can lead to DNA damage in epithelial cells. This occurs via tumor cell toll-like receptors, which trigger the nuclear translocation of the transcription factor nuclear factor-kappa β (NF-kβ) and subsequent cytokine production [44].

Saliva also contains a unique repertoire of ncRNA signatures that can play a crucial role in diagnosing OSCC. Salivary lncRNA analysis and site-specific expression profiles aid in the early identification of OSCC and the tracking of post-surgery recurrence [3,45]. It is important to note that relatively fewer studies have been conducted on ncRNAome, such as lncRNA, circRNA, and miRNA, particularly regarding their clinical utility as non-invasive diagnostic and prognostic markers in saliva samples. Biopsies are a definitive procedure for diagnosing oral malignancy; however, the urgency to identify the disease in its early stages and the requirement to explore its molecular background for effective treatment planning have prompted the development of alternative diagnostic methods [28]. The collection of salivary ncRNAomes has potential as (1) it is less invasive and has no such risks that are associated with the collection of the blood [46], and (2) the collection process of saliva is not only inexpensive and more efficient but can also provide information that is not readily available from serum testing [47]. However, saliva’s individual biomarkers lack the sensitivity and specificity to fulfill rigorous diagnostic standards. The issue of excessively high saliva viscosity arises from mucopolysaccharides and mucoproteins, which can disrupt the analytical procedure. Despite this, saliva is valuable for the early detection, diagnosis, and monitoring of applied therapy for oral cancer [35]. This review, therefore, will primarily examine the salivary ncRNA composition linked to disease progression and investigate their roles as potential biomarkers for early disease detection, aiming to improve survival outcomes among individuals with oral cancer.

## 2. Saliva in Diagnostic Application and the Emergence of Saliva Omics

The significance of saliva in diagnosis is increasingly recognized, and its application as a diagnostic tool for systemic disorders is growing [48]. A trending approach to studying saliva and its components and functions is saliva omics. Saliva omics encompasses biomarkers categorized into genomes, transcripts, proteins, epigenomes, metabolites, and microbiomes [28]. Saliva omics offer a comprehensive method for evaluating diseases, tracking progress, and enabling personalized, non-invasive diagnostic solutions. The study of genomics in saliva provides valuable genetic information from host cells and microorganisms, enabling the analysis of mutations, polymorphisms, and epigenetic changes associated with diseases like oral cancer, thus aiding in risk assessment and personalized medicine [49,50]. Transcriptomics uncovers RNA molecules, including mRNA, miRNA, and lncRNA, that indicate alterations in gene expression due to disease. Specific salivary miRNAs and lncRNAs are associated with conditions such as cardiovascular and autoimmune diseases, facilitating disease monitoring and evaluating treatment responses [51]. Proteomics examines the diverse proteins in saliva, such as enzymes, antibodies, and cytokines. These can reveal oral and systemic health issues, including diabetes and cancer, by identifying inflammatory markers or malignancies for early diagnosis and ongoing disease monitoring. Finally, metabolomics examines small molecules and metabolic byproducts, revealing changes associated with cancer, stress, and metabolic disorders, thus providing insights into alterations in cellular stress and energy metabolism [52].

Multiple research groups have concluded that ncRNAome signatures in saliva represent an attractive, non-invasive, cost-effective strategy for diagnosing and prognosis OSCC [53,54,55,56]. ncRNAs may serve as promising candidates in cancer diagnosis and therapy. This occurs as ncRNAs simultaneously target various druggable and non-druggable targets and signaling events. Additionally, they exhibit tissue specificity, unique RNA characteristics for quick detection, enhanced tissue-related activity, and a significantly more stable structure [57]. In a particular investigation, *miR136*, *miR-147*, *miR1250*, *miR-148a*, *miR632*, *miR646*, *miR668*, *miR877*, *miR503*, *miR220a*, *miR323-5p*, *miR-24*, and *miR-27b* have been determined for diagnostic purposes in patients with OSCC [58,59]. Duz lab found that *miR139-5p* levels were reduced in the saliva of patients with tongue cancer [59,60].

## 3. Crosstalk Between ncRNAome and Oral Microbiota in OSCC

There is increasing evidence that members of the human microbiome are closely linked to a diverse range of cancer types. The microorganisms in the oral cavity can exist as commensals, symbionts, and pathogens [61,62,63,64]. These microbes and their host keep a balanced condition where they are advantageous to one another. During disease states like periodontitis, there may be an increase in the growth of specific commensal microbiota, accompanied by a decline in other oral microbiota within the oral cavity [9,64,65]. The oral microbiome is suggested as a potential diagnostic marker for oral cancer [66]. Various organisms have been identified to increase in OSCC samples, including *Capnocytophaga gingivalis*, *Prevotella melaninogenica*, and *S. mitis* [67]; *F. nucleatum* [65]; *Pseudomonas aeruginosa* [68]; *Campylobacter concisus*, *Prevotella salivae*, *Prevotella loeschii*, and *Fusobacterium* oral taxon 204 [69]; the genera *Fusobacterium*, *Dialister*, *Peptostreptococcus*, *Filifactor*, *Peptococcus*, *Catonella*, and *Parvimonas* [70]; as well as *Prevotella oris*, *Neisseria flava*, *Neisseria flavescens/subflava*, *F. nucleatum* ss polymorphum, *Aggregatibacter segnis*, and *Fusobacterium periodonticum* [71].

Yang’s lab investigated the changes in the microbiome throughout the progression of cancer, from early to late stages, and observed a notable increase in Fusobacteria. They discovered that *F. periodonticum*, *Parvimonas micra*, *Streptococcus constellation*, *Haemophilus influenza*, and *Filifactor alocis* were linked to OSCC at the species level, and their abundance gradually rose from stage 1 to stage 4 [72].

Viruses have historically been linked to the potential development of OSCC [66]. Numerous meta-analyses have shown that infection with human papillomavirus (HPV) increases the possibility of OSCC by as much as three times [73,74]. The average incidence of HPV among OSCC patients is approximately 25% [73,75,76]. Additionally, other viruses have been identified in OSCC samples, either as individual infections or in co-infections with HPV. Nevertheless, it is unclear how they contribute to the disease [76,77,78,79]. Numerous research groups have been investigating the link between the herpes simplex virus (HSV), an adenovirus, and oral cancer in both clinical subjects and animal models [80,81]. A recent investigation has indicated that HSV-1 is dominant in the oral cavity and tumor tissue of OSCC subjects, yet it has no significant impact on OSCC proliferation and invasion [82]. The prevalence of *Candida* is notably high, accounting for 78.8% of all OSCC cases, while *Saccharomyces* represents 76.8% of the OSCC cases, in contrast to a lower presence in healthy controls [83]. The dysbiosis of oral bacteria, resulting from the consumption of broad-spectrum antibiotics, may significantly facilitate the development of hyperplastic Candidiasis, which could lead to OSCC [84]. *Candida* infection induces cytokines like IL8 and TNF-a, which stimulate TLRs that can interact with the NF-kB inflammatory process in oral cancer metastasis [85]. The causality of oral oncogenesis arises from the interaction between the environmental microbiome and the host oral mucosa, influencing a wide range of non-coding RNAs and the host’s tumor immune surveillance and cytotoxicity. Growing evidence connects *Fusobacterium nucleatum* to tumorigenesis [86,87,88]. A research study showed that *F. nucleatum* infection could trigger epithelial-mesenchymal transition (EMT) in oral epithelial cells and outlined a possible signaling pathway that governs the induction of EMT [89]. Further exploration demonstrated the alterations of lncRNA and potential hub genes in oral epithelial cells in response to *F. nucleatum* infection, providing novel insights into the transition from normal to malignant transformation initiated by oral bacterial infection [89]. The analysis results indicate that *LINC00460*, *LINC00511*, *LINC01160*, *LINC00702*, and *MNX1-AS1* may serve as key regulators of the hub genes associated with malignant transformation caused by *Fusobacterium nucleatum* infection [89]. *LINC00460* was identified to exhibit a co-expression pattern with *VEGFA*, which has been demonstrated to be overexpressed in aggressive OSCC [90]. *MNX1-AS1* serves as a natural antisense transcript of *MNX1* and has been identified as a potential oncogenic driver in multiple cancer types [91,92,93]. *MNX1-AS1* overexpression may induce an epithelial–mesenchymal transition (EMT) and stimulate breast cancer’s Akt/mTOR pathway [93]. Further investigation is required to elucidate the role of *MX1-AS1* and *LINC00460* in oral cancer progression. A recent report indicates that the infection of oral epithelial cells with *F. nucleatum* results in elevated expression levels of *MIR4435-2HG*, which can then specifically bind to another non-coding RNA, *microRNA-296-5p*, leading to a downregulation of its expression. A reduction follows this in *mi-296-5p*’s ability to block the expression of its target gene *Akt2*, which subsequently activates the expression of the transcription factor SNAI1 and contributes to the transition into the mesenchymal phenotype of infected oral epithelial cells [94].

Infection with *P. gingivalis* has been positively correlated with advanced clinical staging, poor differentiation, and lymph node metastasis among individuals with OSCC [88,95]. A recent research investigation reveals that the *P. gingivalis* outer membrane vesicles (OMVs) enhance the invasion and proliferation of OSCC cells in vitro [59]. Additional research indicated that *sRNA23392* was prevalent in *P. gingivalis* OMVs and facilitated the invasion and migration of OSCC cells by targeting desmocollin-2 (DSC2) [96]. sRNAs of miRNA size, typically ranging from 15 to 25 nt in length, primarily interact with target mRNAs via incomplete base pairing, influencing the translation and/or stability of the mRNAs [97]. DSC2, a member of the desmosomal cadherin family, has been identified as playing a role in tumor development. *sRNA23392* inhibitors reduced the migration and invasion of OSCC cells induced by *P. gingivalis* OMV [96]. A recent report indicates that *P. gingivalis* may enhance OSCC development by modulating cyclin D1 expression through the *miR-21*/PDCD4/AP-1 negative feedback signaling pathway [95]. The levels of *P. gingivalis* DNA showed a positive correlation with the expression of *miR-21* and *c-Jun* while exhibiting a negative correlation with PDCD4 expression in clinical OSCC samples. The expression of *miR-21* increased as the expression of programmed cell death 4 (PDCD4) decreased [95]. The expression of cyclin miR-21, PDCD4, and AP-1 influenced D1. Blocking *c-Jun*, blocking *miR-21* expression, or overexpressing PDCD4 disrupted the pathway, decreasing cyclin D1 expression and hindering cell proliferation [95,98].

## 4. Targeting Salivary ncRNAome for Oral Cancer Detection

ncRNAs present a promising avenue for OC detection owing to their distinct advantages: (a) Non-Invasive Collection: Saliva collection is convenient and suited for repeated sampling, facilitating early detection and ongoing monitoring [99]. (b) Cancer-Specific Markers: ncRNAomes play a crucial role in regulating gene expression and serve as indicators of tumor growth, metastasis, and treatment resistance, making them a dependable marker for cancer [100]. (c) Local Cellular Reflection: ncRNAs from oral tumor cells enter saliva, directly capturing oral-specific cancer changes. (d) Early Detection: Specific lncRNAs, such as *HOTAIR*, *MALAT1*, and *H19*, exhibit changed levels during the early stages of cancer, facilitating timely intervention [100]. (e) Cost-Effective: Saliva-based lncRNA testing is affordable, particularly with RT-PCR or sequencing, facilitating more accessible routine screening [101].

This review will highlight several seminal works that illustrate the interplay between the expression of specific ncRNA signatures and the progression of OSCC.

## 5. Role of Salivary ncRNAome as Potential Diagnostic Biomarkers

**Figure 1:**
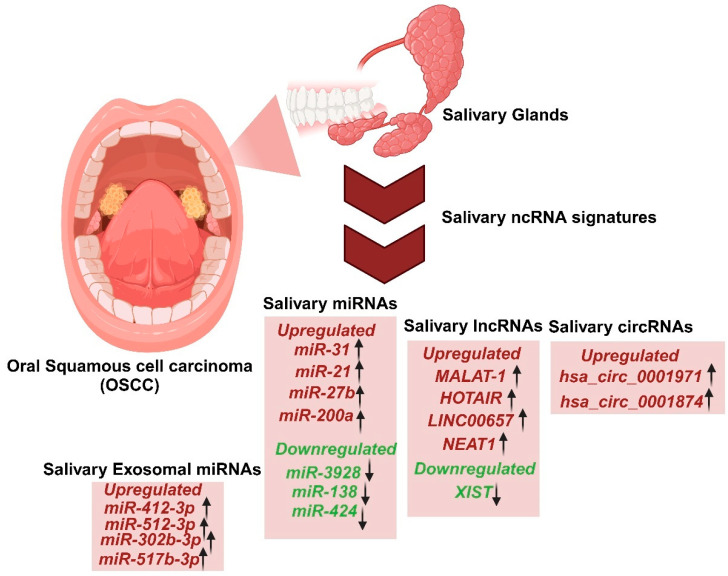
Salivary ncRNAome signatures as diagnostic markers for the detection of oral squamous cell carcinoma (OSCC).

Saliva and other less invasive sampling methods, including oral swabs, brush biopsies, and scrapers, have emerged as practical tools for detecting biomarkers of OSCC. Researchers have discovered several non-coding RNAs (ncRNAs) in saliva that hold clinical significance for OSCC (Table 1, Figure 1). Among these biomarkers, lncRNAs have attracted considerable interest due to their diagnostic and prognostic capabilities.

For instance, it was demonstrated that lncRNAs *HOTAIR* and *MALAT1* are significantly elevated in the saliva of OSCC patients, with higher HOTAIR expression correlating with nodal metastasis, making it a promising non-invasive biomarker for OSCC management [112,113,114]. However, such studies often face limitations, including small sample sizes. These findings underscore the potential of lncRNAs as diagnostic tools but highlight the need for further validation. Salivary miRNAs such as *miR-21*, *miR-31*, *miR-27b* have also shown promise as OSCC biomarkers due to their role in cancer progression [114]. Additionally, Zhao et al. identified dysregulated circRNAs in the saliva of OSCC patients, such as overexpressed *hsa_circ_0001874* and *hsa_circ_0001971*, further expanding the pool of potential diagnostic markers [56].

Despite these advances, further research is needed to validate the diagnostic and prognostic roles of circulatory and salivary lncRNAs and other biomarkers in OSCC. Such studies could pave the way for more effective, non-invasive diagnostic tools and improve the management of OSCC. In the following sections, we will try to explore a few unique ncRNA signatures for a potential biomarker.

### 5.1. Role of Salivary miRNAs as a Biomarker for OSCC

Several pieces of evidence suggest that salivary miRNAs are an up-and-coming method for diagnosing and prognosing OSCC. A careful investigation of these may be crucial in formulating innovative approaches to OSCC treatment.

**Figure 2:**
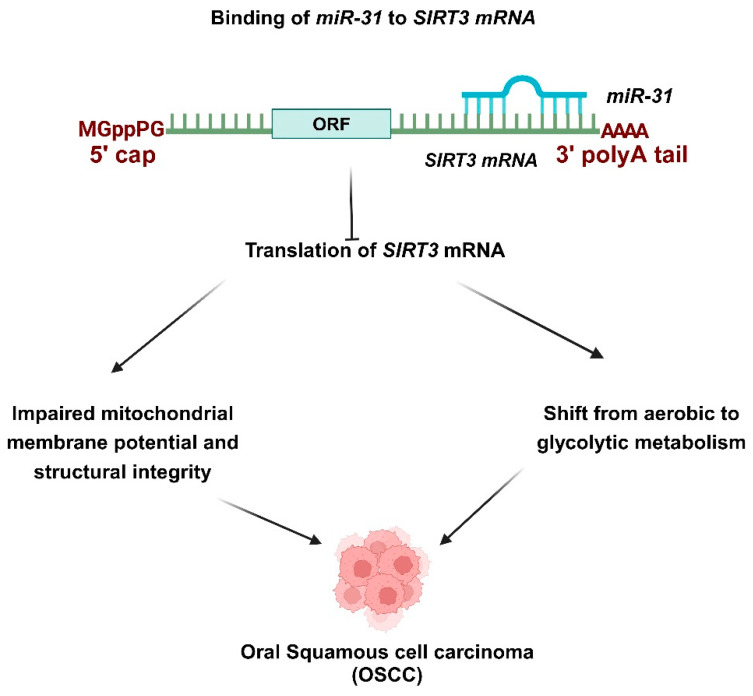
Role of *miR-31* in OSCC progression. *miR-31* targets *SIRT3*, facilitating the invasion of OSCC. OSCC tumors exhibit elevated levels of *miR-31* and reduced expression of *SIRT3*. *miR-31-SIRT3* impaired the mitochondrial membrane potential and structural integrity. This axis’s dysregulation also contributed to the emergence of oxidative stress. Additionally, *miR-31* transformed tumor cells from aerobic metabolism to glycolytic metabolism. *miR-31* has been recognized as upregulated in OSCC and acts as an oncogenic miRNA by targeting *SIRT3*, which disrupts mitochondrial activity. *miR-31* targets *SIRT3*, facilitating the invasion of OSCC. OSCC tumors exhibit elevated levels of *miR-31* and reduced expressions of *SIRT3*. The expression of SIRT3 diminished the tumor cell migration and invasion amplified by miR-31. *miR-31*-SIRT3 impaired the mitochondrial membrane potential and structural integrity. This axis’s dysregulation also contributed to the development of oxidative stress. Additionally, *miR-31* transformed tumor cells from aerobic metabolism to glycolytic metabolism.

*miR-31* is significantly upregulated in saliva compared to plasma, indicating its local contribution to tumorigenesis and its sensitivity in detecting tumors, regardless of size or stage. Notably, levels of *miR-31* diminish after tumor excision, creating a clear connection between its expression and the presence of tumors [115]. Nonetheless, it fails to distinguish OSCC from dysplastic potentially malignant disorders (PMDs), indicating its potential as an early biomarker [116]. *miR-31* has been identified as upregulated in OSCC. It functions as an oncogenic miRNA by targeting SIRT3 thereby disrupting mitochondrial activity (Figure 2) [102]. *miR-31* targets SIRT3 to promote OSCC invasion. OSCC tumors show an increase in *miR-31* levels and a decrease in SIRT3 expression (Figure 2). The expression of SIRT3 reduced tumor cell migration and invasion, which was enhanced by *miR-31* [103]. *miR-31*-SIRT3 hindered the mitochondrial membrane potential and structural integrity. The dysregulation of this axis also played a role in the development of oxidative stress—furthermore, *miR-31* altered tumor cells from aerobic metabolism to glycolytic metabolism [103]. Mitochondrial dysfunction and the adaptation of aerobic glycolysis for energy production are common occurrences in malignancies [102,117]. Conversely, *miR-27b* is upregulated in OSCC saliva, positioning it as a promising diagnostic marker and oncogenic miRNA [118].

*miR-3928* serves as a key regulator in the process of carcinogenesis [119,120]. A study indicates that salivary *miR-3928* functions as a tumor suppressor in OSCC. The expression of *miR-3928* decreased substantially in the OSCC group (67-fold) and the oral potentially malignant disorder (OLP) group (sixfold) as compared to healthy controls. The results indicate that *miR-3928* holds promise as a significant biomarker and may serve as a tumor inhibitor in therapeutic strategies [104]. In a particular study, liquid biopsy assays from saliva revealed that *miR-138* and *miR-424* exhibited decreased expression levels in saliva samples from OSCC and OPMD patients compared to healthy controls. These emerging early diagnostic biomarkers effectively distinguish between OSCC patients, OPMD patients, and healthy subjects [121].

The Arun lab indicated that in OSCC, the dysregulation of the *miR-200* family and EMT-related genes may contribute to tumor metastasis and therapeutic resistance [104,122,123]. Several studies have shown increased salivary *miR-200a* in patients diagnosed with OSCC [123,124,125,126]. Another investigation reveals that the expression levels of two biomarkers, *miR-200* and *miR-34*, are lower in patients than in healthy individuals. In contrast, the expression level of *miR-24* is higher in patients than in healthy individuals [127]. A separate study demonstrated the upregulation of *miR-412-3p*, *miR-512-3p*, *miR-302b-3p*, and *miR-517b-3p* in the extracellular vesicle salivary samples of OSCC patients when compared to healthy controls, highlighting their involvement in OSCC [128,129]. These findings underscore the diagnostic, prognostic, and therapeutic potential of salivary miRNAs in OSCC while highlighting the need for further research to validate their clinical utility.

### 5.2. Role of Salivary lncRNAs as a Biomarker for OSCC

**Figure 3:**
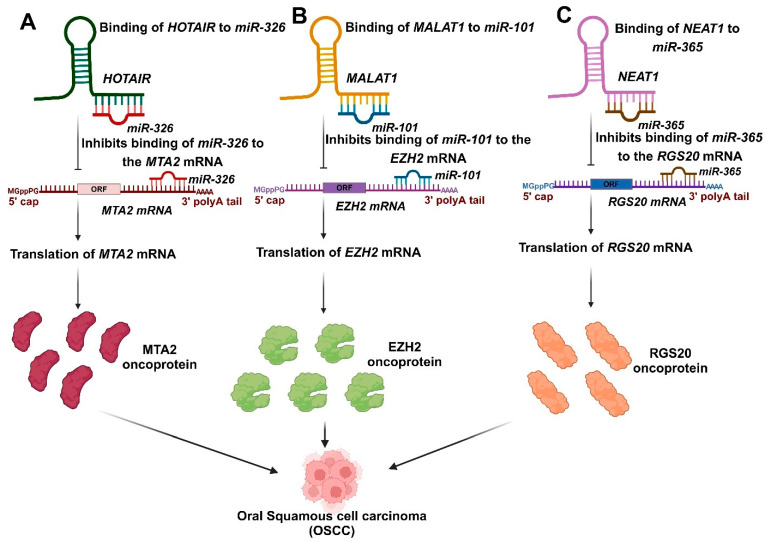
The involvement of salivary lncRNAs in the progression of OSCC. (**A**). The expression of *miR-326* is reduced in OSCC, and the findings indicate that *HOTAIR* acts as a competitive endogenous RNA by sponging *miR-326*, which regulates the derepression of *metastasis-associated gene 2* (*MTA2*). (**B**). *MALAT1* influences the progression of OSCC by negatively regulating *miR-101*, which promotes the expression of *EZH2*. (**C**). *NEAT1* has been identified to enhance proliferation, migration, and invasion by sponging *miR-365*, thereby facilitating the expression of *RGS20* in OSCC.

Salivary lncRNAs have emerged as critical biomarkers in OSCC due to their diverse roles in tumor progression, metastasis, and prognosis. Among them, *HOTAIR* stands out for its significant overexpression in the saliva of OSCC patients with lymph node metastasis, compared to other lncRNAs such as *HULC*, *MALAT-1*, *MEG-3*, *NEAT-1*, and *UCA1* [55]. Metastasis is recognized as the primary factor contributing to mortality in OSCC [130]. Consequently, *HOTAIR* could serve as a potential predictor of patient survival [55]. *miR-326* expression is downregulated in OSCC, and results suggest that HOTAIR functions as a competitive endogenous RNA by sponging *miR-326*, which regulates the derepression of metastasis-associated gene 2 (MTA2) [131] (Figure 3A). MTA2 is a component of the Twist complex that inhibits E-cadherin expression [132]. MTA2 can enhance the epithelial-mesenchymal transition (EMT) and the progression of multiple cancers, negatively impacting their prognosis [133,134]. Overexpression of MTA2 was noted in OSCC cell lines [131].

A research investigation also identified *MALAT1* in saliva and observed no significant difference in the salivary levels of *MALAT1* among metastatic and non-metastatic cases, challenging the established link between *MALAT1* and the metastatic potential of OSCC [55]. The overexpression of *MALAT1* plays a crucial role in governing gene expression, enhancing cell motility, and promoting tumor development as well as epithelial-mesenchymal invasion (EMT). *MALAT1* has been associated with the nuclear accumulation of oncogenes NF-κB, P65, β-catenin, and P-β-catenin, leading to these effects. This resulted in the deregulation of Wnt/β-catenin signaling and an elevated production of MMP-2, MMP-7, and MMP-9 [106,135,136]. Nonetheless, a recent study revealed that OSCC patients exhibited elevated *MALAT1* levels and reduced salivary *miRNA-124* levels compared to normal controls [106]. A recent study indicated that *MALAT1* was upregulated, whereas miR-101 was downregulated in OSCC. *MALAT1* impacts the progression of OSCC through the negative regulation of *miR-101*. Additionally, bioinformatics analysis identified EZH2 as the target of *miR-101*. *MALAT1* promotes EZH2 expression by modulating miR-101, presenting a potential novel target for OSCC treatment [15] (Figure 3B).

Salivary *LINC00657* and *miR-106a* may be effective diagnostic markers for oral squamous cell carcinoma. Salivary *LINC00657* demonstrates an elevated diagnostic accuracy (83.3%) in distinguishing between OSCC grade II and III [54]. In the same way, the Xu lab showed that the overexpression of *LINC00657* was associated with a higher pathological stage, suggesting a generally lower survival rate [108]. The results indicated a reduced expression of salivary *miR-106a* in the OSCC group compared to the OLP and control groups, suggesting that the overexpression of *LINC00657* may be downregulating *miR-106a* expression. Additionally, salivary *miR106a* may be included among the tumor suppressor miRNAs associated with OSCC [54]. Recent findings by Shieh et al. linked the absence of lncRNA *XIST* in saliva to a higher risk of OSCC [137].

In OSCC, *NEAT1* exhibited a significantly higher expression in the saliva of OSCC patients than healthy individuals’ oral mucosa [53,55]. *NEAT1* has been identified to enhance proliferation, migration, and invasion by sponging *miR-365* in OSCC [110]. RGS20 was recognized as a direct target of *miR-365*, and its overexpression hindered the miR-365-induced suppression of OSCC cell proliferation and invasion. RGS20 enhanced cell viability, motility, and the protein expression of cyclin D1 and N-cadherin, while reducing the protein level of E-cadherin, indicating the oncogenic role of RGS20 in OSCC. The regulation of RGS20 protein levels by *NEAT1/miR-365* indicates that *NEAT1* functions as a ceRNA for *miR-365*, thereby promoting the expression of RGS20 [110] (Figure 3C). *NEAT1* also has the potential to enhance proliferation and epithelial-mesenchymal transition (EMT) while inhibiting apoptosis by activating the VEGF-A and Notch signaling pathways in vitro, indicating its role as a regulatory factor in OSCC [109].

The salivary lncRNAs collectively provide essential insights into the progression of OSCC. Their varied mechanisms, including transcriptional regulation and ceRNA activity, highlight the complexity of OSCC pathogenesis and the necessity for additional research to translate these findings into clinical applications.

### 5.3. Role of Salivary circRNAs as a Biomarker for OSCC

There is only limited evidence of salivary circRNAs as diagnostic markers for OSCC. When considered alongside the roles of salivary lncRNAs, circRNAs add another layer of complexity to the molecular landscape of OSCC. Both classes of RNA share mechanisms such as sponging miRNAs and interacting with key signaling pathways, emphasizing their interconnected contributions to tumor progression, metastasis, and treatment resistance. The Bahn lab reported on the prevalence of circRNAs in saliva and their involvement in intracellular signaling cascades in addition to the inflammatory response [128,129,138]. A specific study identified over 400 circRNAs extracted from cell-free saliva in healthy controls [138]. The half-life (t1/2) of circRNA is 48 h, roughly four times longer than that of mRNAs, suggesting enhanced stability [139,140]. The stability, abundance, and favorable half-life of circRNAs indicate their potential as biomarkers [139,141].

**Figure 4:**
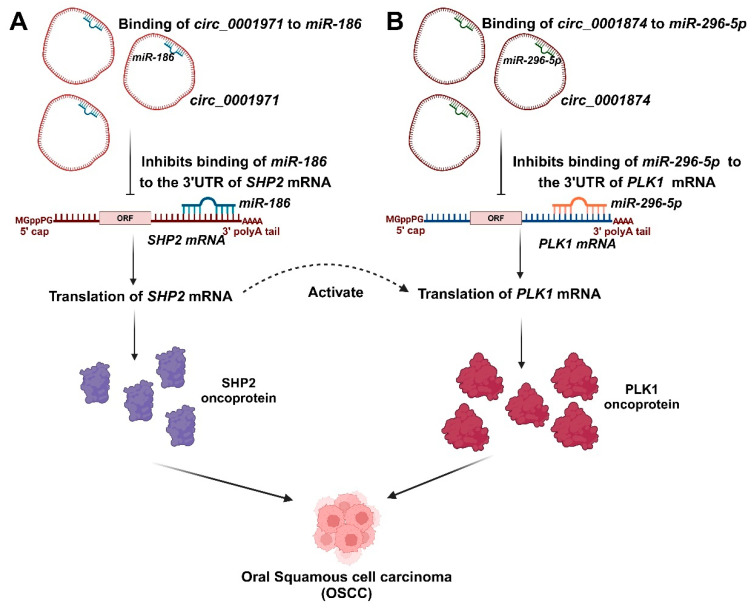
The involvement of salivary circRNAs in the progression of OSCC. (**A**). *Circ_0001971* inhibited the expression of *miR-186*, resulting in the increased expression of the SHP2 oncoprotein. (**B**). *Circ_0001874* inhibited the expression of *miR-296-5p*, resulting in the increased expression of the PLK1 oncoprotein. The dysregulation of *hsa_circ_0001971* may affect *SHP2* and *PLK1*, while *hsa_circ_0001874* influences only *PLK1*. *SHP2* is an essential activator for the oncogenic activity of *PLK1*.

A specific study reported that there were 12 upregulated and 20 downregulated circRNAs in the saliva of OSCC patients compared to healthy controls [56]. In the analysis of differentially expressed circRNAs, *hsa_circ_0001874*, *hsa_circ_0001971*, and *hsa_circ_0008068* showed significant upregulation in the OSCC group compared to the healthy group. Clinical data revealed that salivary *hsa_circ_0001874* was correlated with TNM stage (*p* = 0.006) and tumor grade (*p* = 0.023), while *hsa_circ_0001971* showed a correlation with TNM stage (*p* = 0.019) [56]. Furthermore, the analysis revealed that the expression levels of salivary *hsa_circ_0001874* and *hsa_circ_0001971* were significantly lower in the postoperative samples than in the preoperative samples (*p* < 0.001). Therefore, salivary *hsa_circ_0001874* and *hsa_circ_0001971* may be used as biomarkers to diagnose OSCC [56]. *hsa_circ_0001971* and *hsa_circ_0001874* may exert oncogenic effects, at least in part, via their subsequent signaling networks such as *hsa_circ_0001971/miR-186/SHP2* and *hsa_circ_0001874/miR-296-5p/PLK1*. *MiR-186* has been identified as a tumor suppressor in OSCC, with its downregulation linked to increased expression of the oncogenic factor protein tyrosine phosphatase SHP2 and the activation of growth-promoting signaling pathways [142]. Similarly, *miR-296* functions as a tumor suppressor, and its reduced expression is associated with increased levels of the PLK1 oncogenic factor in non-small cell lung cancer cells [143]. *circ_0001847* and *circ_0001971* inhibited the expression of *miR-296-5p* and *miR-186* through their binding to these miRNAs [111]. Additionally, it was shown that *miR-296-5p* and *miR-186* overexpression could bind to *circ_0001847* and *circ_0001971*, respectively, and significantly lower their levels, suggesting that these circRNAs and miRNAs sponged one another [111]. The two signaling pathways of *circ_0001971/miR-186/SHP2* and *circ_0001874/miR-296-5p/PLK1* govern the oncogenic impacts of *hsa_circ_0001971* and *hsa_circ_0001874* in the progression of OSCC (Figure 4). SHP2 serves as a crucial activator for the oncogenic effect of *PLK1* [144]. The impaired regulation of *hsa_circ_0001971* may influence *SHP2* and *PLK1*, whereas *hsa_circ_0001874* impacts only PLK1 [111].

Collectively, these non-coding RNAs hold promise for advancing our understanding of OSCC pathogenesis and developing novel diagnostic tools and targeted therapies.

## 6. Conclusions

In this review, we specifically focused on the role of salivary ncRNA signatures (including miRNA, lncRNA, and circRNA) in advancing OC (Figure 1). The ncRNAs influence numerous oncogenic and tumor-suppressive pathways, thus influencing the progression of OC. Exploring the role of salivary ncRNAs as potential biomarkers for oral cancer has revealed an intriguing potential for OC detection. Numerous findings have shown that the elevated mortality and morbidity in OC are primarily linked to the challenges of achieving a swift diagnosis and effective management. Consequently, obtaining a timely diagnosis is essential for effectively managing the spread and metastasis of OC, ultimately enhancing the overall life expectancies of patients [145]. Oral dysbiosis, characterized by an imbalance in the oral microbial community, has garnered considerable interest because of its intricate connection to oral cancer. A growing body of evidence connects oral oncogenesis causality to the environmental microbiome and its interaction with the host oral mucosa, which drives the abundance of ncRNA-promoting oral cancer. The comprehensive ncRNA signatures derived from saliva reveal their potential as non-invasive diagnostic and prognostic biomarkers for OC. In our current review, we have observed several molecular signaling mechanisms by which these ncRNA signatures influenced OSCC progression. Further investigation is essential to grasp the mechanisms that underlie OSCC comprehensively. Numerous clinical trials have been carried out involving ncRNAs, particularly focusing on miRNAs as diagnostic and therapeutic biomarkers [146]. A clinical study has commenced to assess both the specificity and sensitivity of *miRNA-412* and *miR-512* in extracellular vesicles derived from saliva regarding the malignant advancement of OC (ClinicalTrials.gov Identifier: NCT04913545) [2]. The significance of lncRNA *MALAT1 and* its target *miR-124* in diagnostics and therapy has been explored in saliva samples from OC patients (ClinicalTrials.gov Identifier: NCT05708209) [2]. A patent (US 20230227914A1) describes a method for detecting head and neck cancer in the oral cavity or throat, including oral squamous cell carcinoma. This involves identifying the amount of expression of two or more miRNAs in a biological sample from a subject. The selected miRNAs include *hsa-let-7a*, *hsa-miR-16*, *hsa-miR-21*, *hsa-miR-451*, *hsa-miR-486-5p*, and *hsa-miR-92a-3p*. In comparison with cancer-free reference samples, the levels of expression of these miRNAs in the biological sample indicate the presence of head and neck cancer of the oral cavity of the throat [98]. Another patent (US20230203493A1) revealed that lncRNA biomarkers are associated with oral squamous cell carcinoma and can be utilized in diagnosing and treating this condition. The biomarkers include lncRNA *RP11-875O11.3*, *LINC01679*, *AP000695.4*, *RP11-339B21.10*, *RP11-426C22.4*, *RP11-426C22.5*, and/or *AP000695.6*. The biomarkers are utilized to develop products aimed at diagnosing oral squamous cell carcinoma. The biomarkers are also utilized in formulating a pharmaceutical composition aimed at managing oral squamous cell carcinoma and another pharmaceutical composition for the same condition [147]. Despite the significant efforts to uncover the basis for detecting mRNA and proteins as biomarkers for diseases, there remains a limited understanding of ncRNAome as the emerging category of biomarkers found in body fluids [148]. However, further extensive pre-clinical research is necessary to effectively incorporate salivary ncRNAome into clinical practice for OC diagnosis. There is an urgent need for an efficient biomarker for OC detection. This is an open question, and further investigations into the salivary ncRNAome in relation to oral cancer will further deepen our understanding of the condition.

## Figures and Tables

**Table 1:** Representative examples of salivary ncRNAs associated with OSCC.

*Salivary microRNAs*			
Name	Level of Expression	Results	References
*miR-31*	Upregulation	The expression of *miR-31* is upregulated in saliva in comparison to plasma, indicating its local contribution to OSCC tumorigenesis. Levels of *miR-31* diminish after tumor excision, creating a clear connection between its expression and the presence of tumors. *miR-31* functions as an oncogenic miRNA by targeting *SIRT3* thereby disrupting mitochondrial activity.	[102,103]
*miR-3928*	Downregulation	Salivary *miR-3928* functions as a tumor suppressor in OSCC. The expression of *miR-3928* decreased substantially in the OSCC group (67-fold) and the oral potentially malignant disorder (OLP) group (6-fold) as compared to healthy controls.	[104,105]
***Salivary lncRNAs***			
*MALAT 1*	Upregulation	OSCC patients exhibit elevated salivary *MALAT1* levels and reduced salivary *miRNA-124* levels in comparison to the normal individuals. MALAT1 impacts the progression of OSCC through the negative regulation of miR-101 promoting EZH2 expression.	[15,106,107]
*HOTAIR*	Upregulation	Significant overexpression of HOTAIR has been observed in the saliva of OSCC patients with lymph node metastasis, compared to other lncRNAs such as HULC, MALAT-1, MEG-3, NEAT-1, and UCA1. miR-326 expression is downregulated in OSCC, and results suggest that HOTAIR functions as a competitive endogenous RNA by sponging miR-326, which regulates the derepression of metastasis-associated gene 2 (MTA2).	[55]
*LINC00657*	Upregulation	Salivary LINC00657 demonstrates an elevated level of diagnostic accuracy (83.3%) in distinguishing between OSCC grade II and III. LINC00657 may play a role in downregulating miR-106a expression.	[54,108]
*NEAT1*	Upregulation	A significantly higher expression of NEAT1 has been observed in the saliva of OSCC patients compared to the oral mucosa of healthy individuals. NEAT1 has been identified to enhance proliferation, migration, and invasion by sponging miR-365. thereby promoting the expression of RGS20 in OSCC. NEAT1 also has the potential to enhance proliferation and epithelial–mesenchymal transition (EMT) while inhibiting apoptosis by activating the VEGF-A and Notch signaling pathways in vitro, indicating its role as a regulatory factor in OSCC.	[55,109,110]
***Salivary circRNAs***			
*hsa_circ_0001874*	Upregulation	hsa_circ_0001874 showed significant upregulation in the OSCC group compared to the healthy group. Salivary hsa_circ_0001874 was correlated with TNM stage (p = 0.006) and tumor grade (p = 0.023). hsa_circ_0001874 may exert oncogenic effects, at least in part via subsequent signaling networks *hsa_circ_0001874/miR-296/PLK1*.	[56,111]
*hsa_circ_0001971*	Upregulation	hsa_circ_0001971 also showed significant upregulation in the OSCC group compared to the healthy group. Salivary hsa_circ_0001971 showed a correlation with TNM stage (p = 0.019). hsa_circ_0001971 may exert oncogenic effects, at least in part via subsequent signaling networks hsa_circ_0001971/miR-186/SHP2.	[56,111]
